# Tumour cell-derived serglycin promotes IL-8 secretion of CAFs in gastric cancer

**DOI:** 10.1038/s41416-024-02735-2

**Published:** 2024-06-11

**Authors:** Xiang Li, Guiping Xie, Jia Chen, Yaohui Wang, Jing Zhai, Lizong Shen

**Affiliations:** 1 Department of Surgical Oncology, Jiangsu Province Hospital of Chinese Medicine, Affiliated Hospital of Nanjing University of Chinese Medicine, Nanjing, 210029 China; 2 Department of General Surgery, the First Affiliated Hospital, Nanjing Medical University, Nanjing, 210029 China; 3 Jiangsu Key Lab of Cancer Biomarkers, Prevention and Treatment, Collaborative Innovation Center for Cancer Personalized Medicine, Nanjing Medical University, Nanjing, 211166 China; 4 Department of General Surgery, Northern Jiangsu People’s Hospital, Yangzhou, 225001 China; 5 Department of Pathology, Jiangsu Province Hospital of Chinese Medicine, Affiliated Hospital of Nanjing University of Chinese Medicine, Nanjing, 210029 China

**Keywords:** Gastric cancer, Gastric cancer

## Abstract

**Background:**

Cancer-associated fibroblasts (CAFs)-derived IL-8 plays important roles in chemoresistance, immunosuppression, and lymph node metastasis of gastric cancer. However, the mechanisms underlying IL-8 production in CAFs remains unclear.

**Methods:**

DNA pulldown assay was performed to identify the transcription factors responsible for IL-8 expression in CAFs, which was further verified using CHIP-qPCR and DNA agarose gel electrophoresis assays. The cellular localisation of IL-8 was analysed using multiplex immunofluorescence (MxIF).

**Results:**

MxIF demonstrated that IL-8 was mainly produced by CAFs in gastric cancer. Lysine[K]-specific demethylase 5B (KDM5B) was identified as an IL-8 transcription factor in CAFs, and the binding of KDM5B to phosphorylated RB1 limited the transcriptional regulation of IL-8 in gastric cancer cells. Serglycin (SRGN) secreted by tumour cells activated the CD44/c-Myc pathway to upregulate KDM5B expression, thereby promoting IL-8 production in CAFs. Furthermore, tumour-associated neutrophils (TANs)-derived regenerating family member 4 (REG4) upregulates SRGN expression by activating cAMP-responsive element binding protein 1 (CREB1) in gastric cancer cells. Thus, the SRGN-IL-8-TANs-SRGN loop, which facilitates tumour progression, has been explored in gastric cancer.

**Conclusions:**

This study revealed the mechanisms of the preferential production of IL-8 by CAFs in gastric cancer, and paves the way for potential new therapeutic strategies for gastric cancer.

## Background

Gastric cancer remains one of the most common malignancies globally, although its incidence has been declining gradually over the past several decades in several countries [1–3]. Its initiation and progression are complicated and still elusive, resulting in few advances in novel therapy other than radical gastrectomy or chemotherapy [4, 5]. The prognosis is also unsatisfactory [6]. The tumour microenvironment (TME) comprises various cells in addition to tumour cells, such as stromal cells, lymph cells and myeloid cells. Each component and crosstalk *via* several kinds of soluble molecules play important roles in tumour progression and therapeutic responses [7, 8]. Profound research on TME may shed light on the underlying mechanisms of gastric cancer, which helps develop new strategies and improve patient prognosis.

Previously, we investigated the role of cancer-associated fibroblasts (CAFs), one of the predominant stromal cells, in gastric cancer. *H. pylori* infection increases vascular adhesion molecule 1 (VCAM1) levels in CAFs, and VCAM1 subsequently promotes tumour invasion *via* interaction with integrin αvβ5 on the tumour cell surface [9]. CAFs-derived hyaluronan and proteoglycan link protein 1 (HAPLN1) can remodel the extracellular matrix, and facilitates tumour invasion in gastric cancer [10]. Importantly, we showed that CAFs are the principal source of serum IL-8 in gastric cancer, and that CAFs-derived IL-8 promotes chemoresistance to cisplatin *via* activating NF-κB and up-regulating ABCB1 expression [11]. Our retrospective study revealed that elevated serum IL-8 levels can predict lymph node metastasis (LNM) and tumour recurrence, suggesting a potential biomarker for gastric cancer [12]. Furthermore, elevated serum IL-8 levels increase PD-1 expression in CD8^+^ T cells, resulting in an immunosuppressive niche in the primary tumour and tumour-draining lymph nodes (TDLNs), which enhances LNM of gastric cancer [13]. Collectively, our study demonstrated that CAFs, especially CAFs-derived IL-8, play crucial roles in gastric cancer. However, the mechanisms underlying the preferential production of IL-8 by CAFs in gastric cancer remain unclear.

In the current study, we investigated the interactions between tumour cells, CAFs, and tumour-associated neutrophils (TANs) in the gastric cancer microenvironment, identified lysine[K]-specific demethylase 5B (KDM5B) as the transcription factor of IL-8 in CAFs, and demonstrated that tumour cell-derived serglycin (SRGN) mediated by TANs promotes IL-8 production *via* upregulating KDM5B expression in CAFs. Our study revealed the mechanisms of IL-8 production and the instrumental roles of stromal cells in gastric cancer, which may pave the way for potential new therapeutic strategies for gastric cancer.

## Materials and Methods

### Human peripheral blood and tissue specimens of gastric cancer patients

Peripheral blood and tissue specimens were collected from gastric cancer patients with informed consent at the Department of Surgical Oncology, Affiliated Hospital of Nanjing University of Chinese Medicine. All the patients were pathologically diagnosed with primary gastric adenocarcinoma and did not receive preoperative chemotherapy or radiotherapy. The preoperative peripheral blood specimens were collected. Tumour tissues and corresponding noncancerous mucosa tissues (at least 5 cm from the outer tumour margin) were collected immediately after resection, and snap frozen in liquid nitrogen for further analysis. This study was approved by the Institutional Review Board of Nanjing University of Chinese Medicine and conducted in accordance with the principles of the Declaration of Helsinki.

### Isolation and culture of CAFs and normal fibroblasts (NFs)

Isolation and culture of CAFs and normal fibroblasts (NFs) were performed as previously described [9, 10]. In brief, primary CAFs were isolated from gastric carcinoma tissue samples, and primary NFs were isolated from the noncancerous mucosa tissues at least 5 cm from the outer tumour margin in the same patient. Fresh samples were washed with serum-free DMEM, cut into small pieces, and were transferred to a 0.15% collagenase IV solution, followed by incubation at 37 °C for 40 min. Digested cells were filtered through a 40-mm cell strainer (Milex-GP) and centrifuged at 1500 rpm for 10 min. The single-cell suspension was incubated in a Fibroblast Medium Kit (Cat. No. P60108, Innoprot) for 24 h, allowing fibroblasts to attach on culture plates. Unattached cells were removed after 24 h incubation, and the adherent cells were further cultivated for experiments. Cultured CAFs and NFs less than five passages were used for the experiments.

### DNA pulldown assay

The DNA pulldown assay was based on the binding ability of desulfobiotin-containing probes with streptavidin beads. When DNA beads are incubated with cellular proteins, DNA-protein complexes are attached to the beads and purified to obtain the associated proteins. DNA probes were synthesised by polymerase chain reaction (PCR) based on the IL-8 promoter sequence (F: 5’-CTATCCGGCCCAAGCTTT-3’; R: 5’-AATAATTCACCTTGGTGTAAC-3’). DNA probes were labelled with Biotin-11-dUTP and were recollected using VAHTS DNA Clean Beads (Vazyme, Nanjing, China). CAFs were collected after trypsin digestion and nucleoproteins were purified using NE-PER Nuclear and Cytoplasmic Extraction Reagents (Thermo Fisher, MA, USA). The nuclear protein extract (70 μg) was mixed, and incubated with DNA probes (1 μg) and Bioeast Magnetic Streptavidin (Mag-SA) Beads (Bioeast Mag-SA, Hangzhou, China) (50 μl) at 4 °C for 1 h. Then, the mixtures were centrifuged at 5000×g for 30 s, and the precipitate were washed three times with ice-cold PBS, resuspended in 30 μl of loading buffer, and boiled at 100 °C for 5 min. The collected samples containing the bound proteins were processed by SDS-PAGE for further silver staining and liquid chromatograph mass spectrometer (LC-MS) analysis.

### Chromatin Immunoprecipitation (ChIP)

ChIP assays were performed using the SimpleCHIP® Enzymatic Chromatin IP Kit (Cell Signalling Technology, MA, USA) according to the manufacturer’s recommended protocols. Cell lysates of CAFs and AGS (4 × 10^7^ cells) were prepared, and chromatin fragments were fragmented to an average size of 150-900 bp by microcapsule nuclease, and enriched with magnetic beads coated with the antibodies of KDM5B or phosphorylated cAMP responsive element binding protein 1 (p-CREB1) and isotype IgG. Then, the concentrated sample was crosslinked with the input DNA, and the DNA was purified with sodium chloride and protease K. Finally, the specific sequences from immunoprecipitated and input DNA were determined by real-time quantitative PCR (qPCR) for the upstream of IL-8 and SRGN promoter regions. The four primer pairs for the IL-8 and SRGN promoter regions used in qPCR analyses are listed in supplementary Table s1. Pair #3 for IL-8 and pair #2 for SRGN were used for this assay. The qPCR products were processed on 2% agarose gels and visualised on a UV transilluminator.

### Immunohistochemistry assay (IHC)

Immunohistochemistry (IHC) assays were performed *as per* standard protocols. The monoclonal antibodies used were mouse anti-KDM5B (ab244220, Abcam, Cambridge, UK), mouse anti-retinoblastoma 1 (RB1) (anti-RB1) (#9309T, Cell Signalling Technology, MA, USA), rabbit anti-phosphorylated RB1 (anti-pRB1) (#8516 T, Cell Signalling Technology, MA, USA), rabbit anti-SRGN (A6951, Abclone, Wuhan, China), rabbit anti-CD44 (ab189524, Abcam, Cambridge, UK), rabbit anti-CREB1 (#9197, Cell Signalling Technology, MA, USA), and rabbit anti-pCREB1 (ab32096, Abcam, Cambridge, UK).

### Multiplex-immunofluorescence (MxIF) and immunofluorescence

Multiplex-immunofluorescence staining was performed following the manufacturer’s recommended protocols using the Opal 7-Colour Automation Detection IHC Kit (NEL811001KT, PerkinElmer, MA, USA). Consecutive staining rounds included α-SMA (#19245, Cell Signalling Technology, MA, USA), MPO (Ab208670, Abcam, Cambridge, UK), CD68 (#76437, Cell Signalling Technology, MA, USA), IL-8 (ab18672, Abcam, Cambridge, UK), CD3 (#85061, Cell Signalling Technology, MA, USA), and pan-CK (#4545, Cell Signalling Technology, MA, USA). Immunofluorescence was performed with α-SMA (#19245, Cell Signalling Technology, MA, USA) and KDM5B (ab244220, Abcam, Cambridge, UK).

### Western blotting assay

The expression of the indicated protein was determined by western blotting. The antibodies included mouse anti-KDM5B (ab244220, Abcam, Cambridge, UK), rabbit anti-H3K4me3 (#9751, Cell Signalling Technology, MA, USA), mouse anti-RB1 (#9309 T, Cell Signalling Technology, MA, USA), rabbit anti-pRB1 (#8516 T, Cell Signalling Technology, MA, USA), rabbit anti-SRGN (A6951, Abclone, Wuhan, China), rabbit anti-CD44 (ab189524, Abcam, Cambridge, UK), rabbit anti-CREB1 (#9197, Cell Signalling Technology, MA, USA), rabbit anti-pCREB1 (ab32096, Abcam, Cambridge, UK), rabbit anti-regenerating family member 4 (anti-REG4) (ab255820, Abcam, Cambridge, UK), rabbit anti-SFPQ (ab177149, Abcam, Cambridge, UK), rabbit anti-SAP30 (ab231804, Abcam, Cambridge, UK), and rabbit anti-c-Myc (#18583, Cell Signalling Technology, MA, USA). Relative levels were quantified and normalised to β-actin levels in the same sample using density analysis.

### Enzyme-linked Immunosorbent Assay (ELISA)

After treatment with SRGN (MedChemExpress, NJ, USA), GSK467 (MedChemExpress, NJ, USA), or CMs of tumour cells, cell supernatants were collected at the indicated times, and centrifuged for ELISA assay using a human IL-8 ELISA Kit (EH005-96, ExcellBio, China). The assay was performed according to the manufacturer’s instructions. Each experiment was repeated at least thrice.

### Cell culture and treatment

The gastric cancer cell lines AGS, HGC27, MKN45, and MKN28, and the human stomach fibroblast line Hs738, were preserved in our group. All cells were authenticated and mycoplasma was negative. All the cells were cultured in DMEM (Invitrogen, Carlsbad, CA, USA) with 10% FBS (Gibco, Grand Island, NY, USA) and 1% penicillin/streptomycin (Thermo Scientific, Waltham, MA, USA) in a humidified incubator at 37 °C with 5% CO_2_.

### Co-culture of neutrophils and tumour cells

Co-culture of neutrophils and tumour cells were performed according to our previous report [14]. Peripheral neutrophils were isolated from the blood samples of healthy donors or patients with gastric cancer using a human peripheral blood neutrophils separation reagent kit (Solarbio, Beijing, China) according to the recommended protocols. The cells were washed with red blood cell lysis buffer (BD Biosciences, NJ, USA), centrifuged, and washed with PBS. For co-culture of neutrophils and tumour cells, in brief, approximately 2 × 10^5^ gastric cancer cells were seeded in the bottom of 6-well plate culturing for 48 hours. Then approximately 3 × 10^5^ neutrophils isolated and purified from healthy donors were seeded in the upper chamber. After co-cultured for 6 hours, neutrophils and corresponding tumour cells were collected for further analysis.

### RNA isolation and real-time quantitative PCR (qPCR)

Total RNA was extracted and analysed using qPCR. The relative gene expression was normalised to that of GAPDH. Specific primer sets used for this assay included SRGN (F: 5’-AGGTTATCCTACGCGGAGAG-3’, R: 5’-GTCTTTGGAAAAAGGTCAGTCCT-3’), REG4 (F: 5’-TGAGGAACTGGTCTGATGCCGA-3’, R: 5-’TCCATATCGGCTGGCTTCTCTG-3’) and GAPDH (F: 5’-TTGCCATCAATGACCCCTTCA-3’, R: 5’-CGCCCCACTTGATTTTGGA-3’).

### Lentivirus infection

Lentiviruses carrying KDM5B, KDM5B^H499Y^, RB1, KDM5B short hairpin RNA (shKDM5B), and RB1 short hairpin RNA (shRB1) were constructed by GenePharma Co., Ltd. (Shanghai, China). Transduction was performed according to the manufacturer’s instructions. The shRNAs targeting the sequences for KDM5B were CGAGATGGAATTAACAGTCTT, and CCACATTATTTCTAGTCCAAA for RB1.

### Clone formation assay

A total of 800 indicated cells were seeded in 6-well plates, and cultured for approximately 14 d. The cells were fixed with 70% methanol and stained with Giemsa solution. Colonies containing more than 50 cells were considered to be survivors.

### Cell invasion assay

The cell invasion assay was performed in a 24-well Transwell Chamber (Costar, Corning, NY, USA) coated with Matrigel (BD Pharmingen, San Jose, CA, USA). The indicated cells (2 × 10^5^/200 μl) were cultured in the upper chamber in serum-free medium with 10% FBS medium in the lower compartment. After incubation at 37 °C for 24 h, the cells were fixed with 4% paraformaldehyde, stained with crystal violet, and then photographed under a microscope.

### Wound-healing assay

The indicated cells were seeded and cultured in 6-well plates until a confluent monolayer was formed. A sterile plastic tip was used to scratch the monolayer. Images were taken using a microscope at specified time points to observe the migration distance. Migration was quantified as the percentage of wound closure.

### Establishment of subcutaneous allograft tumour model of gastric cancer in Balb/c nude mice

Animal studies were approved by the Animal Management and Use Committee of Nanjing University of Chinese Medicine. Male Balb/c nu/nu mice (SPF, 4 weeks) were purchased from the Institute of Biomedical Sciences, Nanjing University. Sixteen mice were randomly divided into two groups. Approximately 5 × 10^6^ AGS-con or AGS-shKDM5B cells were subcutaneously injected into the nude mice. The mice were euthanized and the tumour tissues were harvested after 3 weeks. Tumour volume was calculated as width × length × (width + length) / 2.

### Statistical Analysis

Statistical analysis was performed using the GraphPad Prism software (version 8.0; La Jolla, CA, USA). All values are expressed as the mean ± standard error. Differences between groups were compared using a two-tailed unpaired Student’s t-test or ANOVA for comparison of two or multiple groups, respectively. Categorical variables were assessed using the chi-square test. Overall survival was assayed using the Kaplan-Meier method and the Gehan-Breslow-Wilcoxon test. All experiments were repeated at least three times, and *P* < 0.05 was considered significant.

## Results

### KDM5B transcriptionally regulates IL-8 expression in CAFs

Previously, we demonstrated that IL-8 is mainly generated by CAFs, and plays an important role in gastric cancer progression. It is closely related to chemotherapy resistance, lymph node metastasis, and poor prognosis [11–13]. However, the exact mechanisms underlying IL-8 production by CAFs remain unclear.

**Fig. 1:**
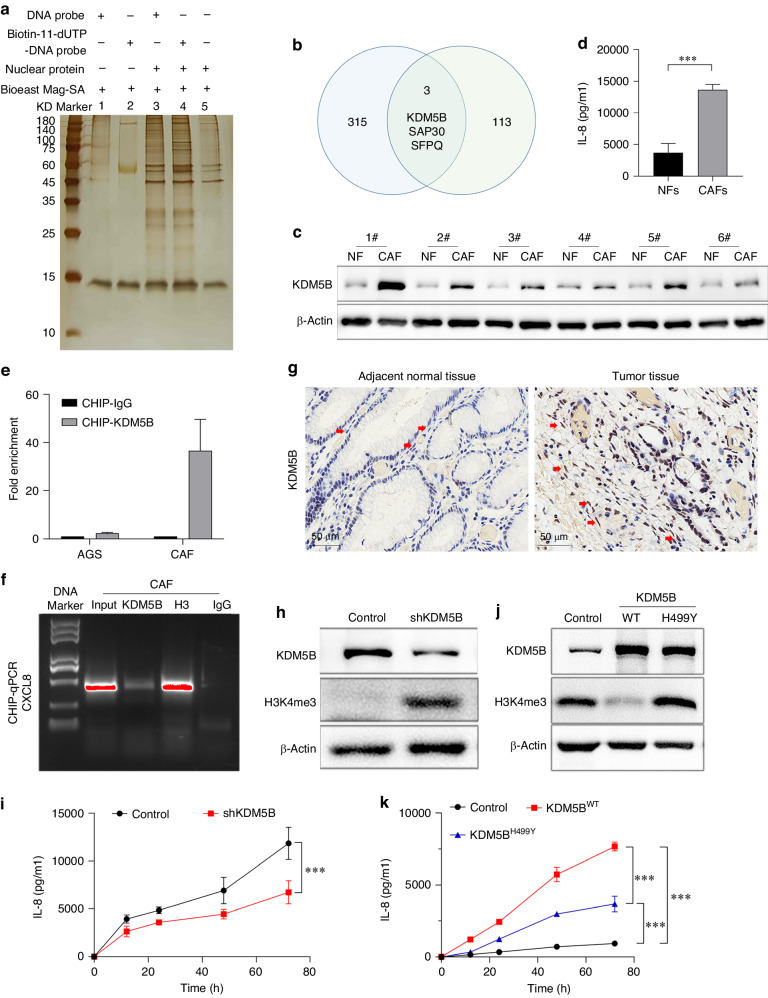
KDM5B transcriptionally regulates IL-8 expression in CAFs. **a** Potential IL-8 promoter-binding proteins in nuclear protein extracts of CAFs, highly expressing IL-8, were pulled down. SDS-PAGE and silver staining showed that the target proteins showed a significant difference between band 3 and 4. **b** DNA-pulldown assay showed 116 proteins were observed to have the potential to bind to the IL-8 promoter region, and searching with the tumour-related transcription factor library indicated that three proteins, KDM5B, SAP30, and SFPQ, were highlighted. **c** KDM5B was upregulated in CAFs with regard to NFs, which was consistent with IL-8 levels (**d**). **e** The CHIP-qPCR indicated that KDM5B could directly bind to the promoter region of IL-8 in CAFs, but it could not occur AGS cells. **f** The PCR products were processed on 2% agarose gels and visualised on a UV transilluminator, showing that KDM5B could directly bind to the promoter region of IL-8 in CAFs. **g** IHC analysis showed that KDM5B levels in CAFs (tumour tissue, red arrow) were significantly higher than in NFs (adjacent normal tissue, red arrow). **h**, **i** KDM5B silence with shRNA resulted in the reduction of IL-8 production in CAFs. **j** KDM5B^WT^ transfection significantly reduced the H3K4 tri-methylation level, but KDM5B^H499Y^ transfection did not. **k** Both KDM5B^WT^ and KDM5B^H499Y^ transfection could significantly increase the secretion of IL-8 in Hs738 cells, and IL-8 level in KDM5B^WT^-transfected cells was much higher than in KDM5B^H499Y^-transfected cells. (***P < 0.001).

Therefore, we performed a DNA pulldown assay to identify the transcription factors responsible for IL-8 expression in CAFs. SDS-PAGE and silver staining indicated that the target proteins in Biotin-11-dUTP-labelled DNA probes showed significant differences with regard to in unlabelled DNA probes (Fig. 1a). Further LC-MS analysis showed 116 proteins had the potential to bind to the IL-8 promoter region. A search of the tumour-related transcription factor library (http://cistrome.org/) indicated that three proteins, KDM5B, SAP30, and SFPQ, were highlighted among these 116 proteins (Fig. 1b). Further assays showed that only KDM5B was upregulated in CAFs compared to NFs, which was consistent with the IL-8 levels (Figs. 1c, d, s1a, b, c). Thus, KDM5B was identified for subsequent studies. ChIP-qPCR (Fig. 1e) and agarose gel electrophoresis (Fig. 1f) assays indicated that KDM5B could directly bind to the promoter region of IL-8 in CAFs, but not in the gastric cancer cell line AGS. IHC analysis of gastric tumours and adjacent normal tissues further proved that KDM5B levels in CAFs were significantly higher than those in NFs (Fig. 1g). Immunofluorescence assays with gastric tumour tissues also showed that KDM5B was mainly expressed in most of CAFs (Fig. s1d). Furthermore, KDM5B silencing with shRNA (Fig. 1h, i) or treatment with its inhibitor, GSK467 (Fig. s1e), significantly reduced IL-8 production in CAFs. These results indicate that KDM5B directly regulates IL-8 expression in CAFs.

KDM5B regulates gene transcription and cell differentiation by modulating H3K4 methylation levels [15]. To ascertain whether the H3K4 demethylase activity of KDM5B is related to IL-8 expression, two lentivirus vectors containing the wild-type KDM5B plasmid (KDM5B^WT^) or its H499Y mutation plasmid (KDM5B^H499Y^) were constructed. KDM5B^H499Y^ abrogates the demethylase activity of KDM5B [16]. The two plasmids were transfected into Hs738 cells, respectively. H3K4 tri-methylation levels were assayed. KDM5B^WT^ transfection significantly reduced H3K4 tri-methylation levels, whereas KDM5B^H499Y^ transfection did not affect its level (Fig. 1j). Moreover, both KDM5B^WT^ and KDM5B^H499Y^ transfection significantly increased the secretion of IL-8 in Hs738 cells, and IL-8 levels in KDM5B^WT^-transfected cells were much higher than those in KDM5B^H499Y^-transfected cells (Fig. 1k). These findings suggested that KDM5B H3K4 demethylases activity is involved in the regulation of IL-8 transcription.

### RB1 inhibits IL-8 transcription in gastric cancer cells

**Fig. 2:**
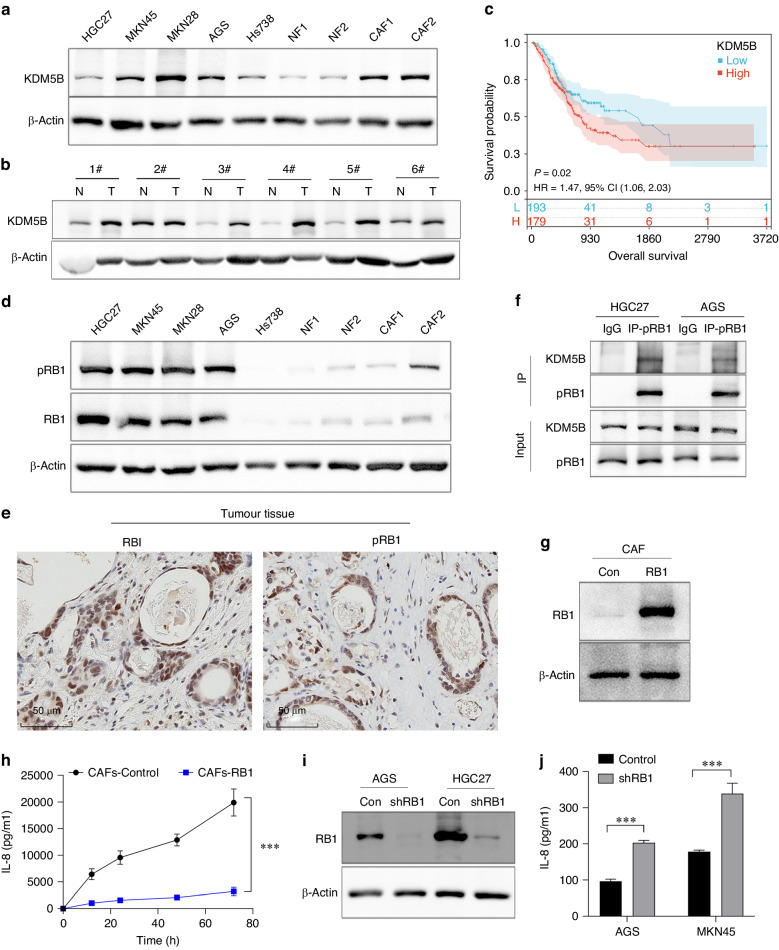
RB1 inhibits IL-8 transcription in gastric cancer cells. **a** KDM5B was detected in gastric cancer cell lines, HGC27, MKN45, MKN28 and AGS cells, as well as in Hs738 and primary CAFs or NFs. **b** KDM5B level in stomach cancer tissues (T) was much higher than in adjacent normal mucosa tissues (N). **c** Analysis with TCGA database showed that the higher levels of KDM5B in stomach tumour tissues were significantly associated with poor prognosis of patients (*P* = 0.020). **d** RB1 was phosphorylated in gastric cancer cells, and pRB1 was hardly expressed in fibroblasts. **e** IHC showed RB1 and pRB1 were highly expressed in tumour cells. **f** Co-IP showed that KDM5B could bind with pRB1in gastric cancer cells. **g**, **h** RB1 expression was upregulated in CAFs, and IL-8 secretion was significantly inhibited. **i**, **j** RB1 silence with shRNA in gastric tumour cells resulted in increased IL-8 levels. (***P < 0.001).

The aforementioned IHC assays with human stomach adenocarcinoma revealed that KDM5B was also detected in tumour cells, in addition to CAFs (Fig. 1g). Further assays showed that KDM5B was detected in gastric cancer cell lines HGC27, MKN45, MKN28, and AGS, as well as in Hs738 and primary CAFs and NFs (Fig. 2a). However, our previous studies and the current multiplex immunofluorescence (MxIF) indicated that IL-8 was mainly expressed in CAFs rather than in tumour cells (Fig. s2a), as did real-time quantitative PCR (qPCR) and ELISA assays (Fig. s2b, c). It has been reported that KDM5B is involved in tumour proliferation, metastasis, and immune evasion [17–19]. We showed that KDM5B levels in stomach cancer tissues were much higher than those in adjacent normal mucosa tissues (Figs. 2b, s3a), which was consistent with the analysis of the TCGA database (Fig. s3b). Furthermore, higher levels of KDM5B in stomach tumour tissues were significantly associated with a poor prognosis (*P* = 0.020) (Fig. 2c). Accordingly, KDM5B knockdown with shRNA (Fig. s3c) significantly suppressed gastric cancer cell proliferation, invasion, and migration in wound-healing (Fig. s3d, e), transwell (Fig. s3f, g), and clone formation assays (Fig. s3h, i). Furthermore, the in vivo study in Balb/c nude mice also showed that KDM5B silence with shRNA suppressed tumour cell proliferation (*P* < 0.001) (Fig. s3J).

Next, we investigated the underlying mechanisms for the difference of IL-8 production between gastric tumour cells and CAFs, although both expressed KDM5B. It has been reported that KDM5B regulates gene transcription *via* binding to phosphorylated RB1 (pRB1) [20]. As a tumour suppressor gene, RB1 is functionally compromised in many tumours owing to mutation, deletion or phosphorylation inactivation [21]. Interestingly, we showed that RB1 was more prone to phosphorylation in gastric cancer cells, and pRB1 was hardly expressed in fibroblasts (Fig. 2d). IHC assays of stomach cancer tissues also showed that RB1 and pRB1 were highly expressed in the tumour cells (Fig. 2e). Co-IP experiments showed that KDM5B could bind to pRB1 in gastric tumour cells (Fig. 2f). Therefore, we speculated that the failure to produce IL-8 in gastric tumour cells may be attributed to the competitive binding of the KDM5B transcription site by pRB1. We upregulated RB1 expression in CAFs using a lentiviral infection system (Fig. 2g), and found that IL-8 secretion was significantly inhibited (Fig. 2h). Furthermore, RB1 silencing with shRNA in gastric tumour cells resulted in increased IL-8 levels (Fig. 2i, j). Collectively, these results indicate that binding of KDM5B to pRB1 limits the transcriptional regulation of IL-8 expression in gastric tumour cells.

### Gastric cancer cell-derived SRGN is associated with poor prognosis and IL-8 secretion

**Fig. 3:**
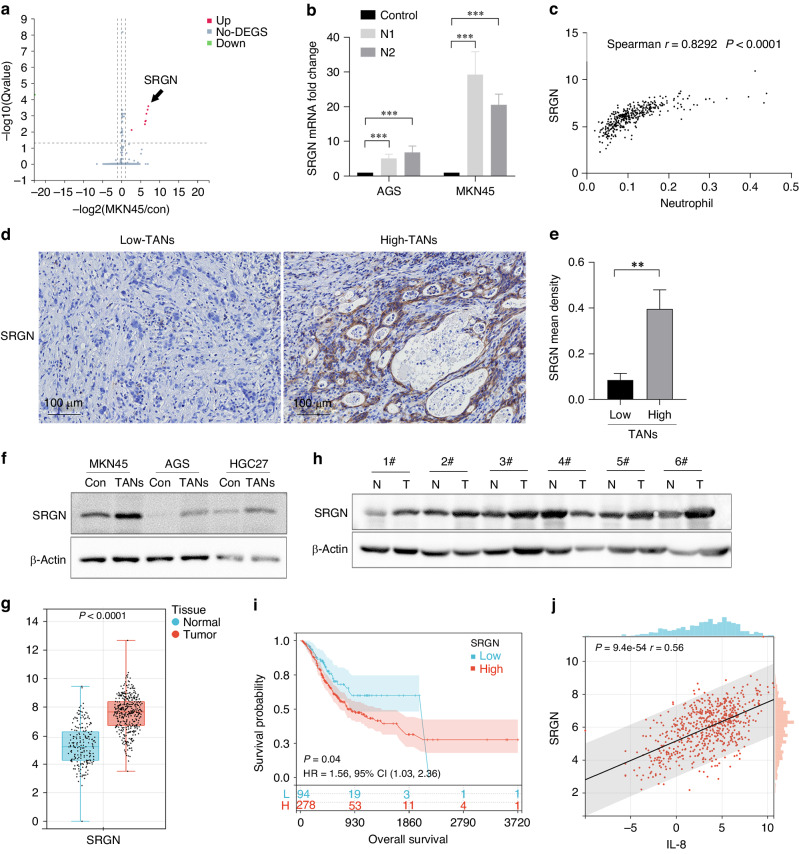
Gastric cancer cell-derived SRGN is associated with poor prognosis and IL-8 secretion. **a**, **b** Co-cultured with neutrophils leaded to multiple variants in tumour cells, and SRGN was one of the most upregulated genes, which was verified using qPCR assays (N, neutrophils). **c** Analyses with TCGA database showed that SRGN level is significantly correlated with neutrophils infiltration in gastric cancer. **d**, **e** IHC assays showed that SRGN was highly expressed in tumour tissues with more neutrophils (High-TANs) than in Low-TANs group (*P* < 0.010). **f** Treatment with CMs of TANs from patients with locally advanced gastric cancer upregulated SRGN levels in gastric cancer cell lines. **g** Analyses with TCGA database showed that SRGN levels in gastric cancer tissues are significantly higher than in adjacent normal tissues (*P* < 0.010), and assays with clinical specimens also obtained similar results (**h**). **i** SRGN levels in tumour tissues are closely associated with poor prognosis of gastric cancer patients (*P* = 0.040). **j** SRGN levels in gastric cancer tissues were positively correlated with the IL-8 levels (*P* < 0.001). (**P < 0.01; ***P < 0.001).

However, the mechanisms underlying KDM5B up-regulation in CAFs remain unclear. As a proinflammatory chemokine, IL-8 recruits neutrophils into the TME. Infiltrated neutrophils, namely tumour-associated neutrophils (TANs), play an important role in tumour progression [22]. Previously, we demonstrated that crosstalk between neutrophils and tumour cells is required for enhanced tumour cell invasiveness, and that neutrophils boost the epithelial-to-mesenchymal transition (EMT) of tumour cells *via* secreting FAM3C [14]. Furthermore, we showed that co-culture with neutrophils resulted in multiple variants of tumour cells. According to RNA sequencing results, serglycin (SRGN) was one of the most upregulated genes (Fig. 3a), as verified by qPCR (Fig. 3b). Herein, analyses using TCGA database showed that SRGN levels were significantly correlated with neutrophil infiltration in gastric cancer (Fig. 3c), and IHC assays with gastric cancer tissues showed that much more immunoreactivity of SRGN was detected in tumour tissues with more neutrophils (high-TANs) than in the low-TANs group (*P* < 0.010) (Fig. 3d, e). Treatment with conditioned media (CMs) of TANs from patients with locally advanced gastric cancer also upregulated SRGN levels in the gastric cancer cell lines (Fig. 3f). Analyses using TCGA database showed that SRGN levels in gastric cancer tissues were significantly higher than those in adjacent normal tissues (*P* < 0.010) (Fig. 3g), and assays with clinical specimens also obtained similar results (Figs. 3h, s4a). In addition, SRGN levels in tumour tissues were closely associated with the clinical stage (Fig. s4b), and poor prognosis of patients with gastric cancer (*P* = 0.040) (Fig. 3I). These results clearly indicate that tumour cell-derived SRGN is involved in gastric cancer progression.

Furthermore, bioinformatics analyses showed that SRGN-related genes were mainly enriched in neutrophil activation, immune regulation, and inflammatory responses (Fig. s4c), and that SRGN levels in gastric cancer tissues were positively correlated with IL-8 levels (*p* < 0.001) (Fig. 3j). Thus, it was reasoned that tumour cell-derived SRGN might be involved in IL-8 production in the gastric cancer microenvironment.

### SRGN upregulates KDM5B expression in CAFs

**Fig. 4:**
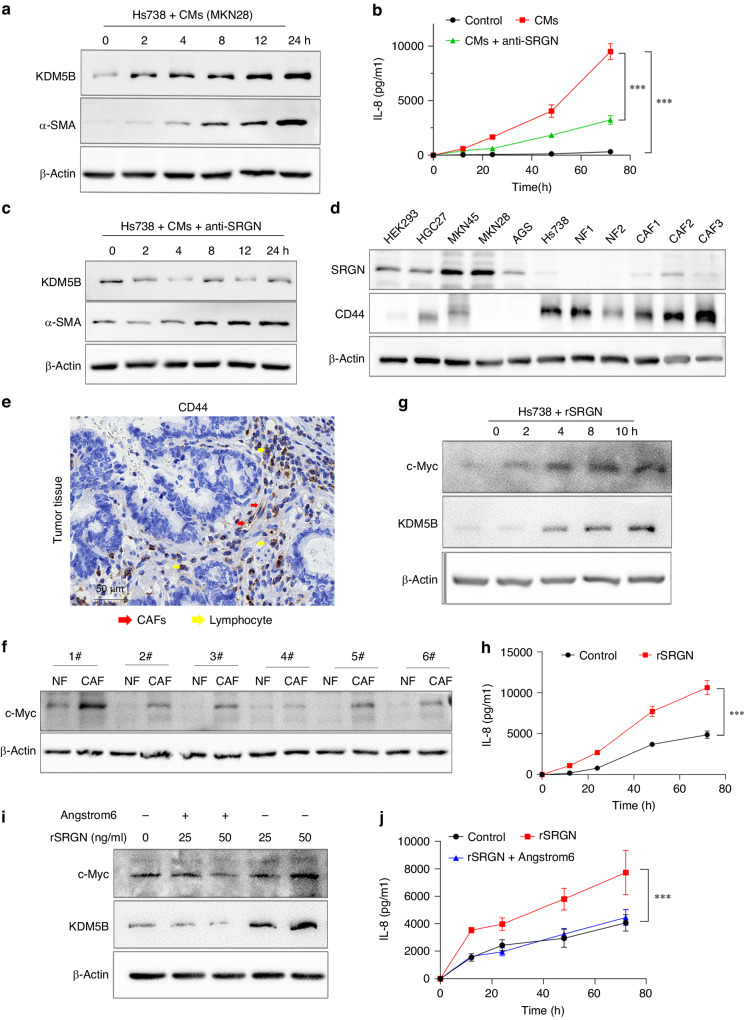
SRGN upregulates KDM5B expression in CAFs. **a** Treatment with CMs of tumour cells increased α-SMA and KDM5B levels in Hs738 cells, and enhanced IL-8 levels (**b**). **c** Addition of anti-SRGN antibody could inhibit KDM5B up-regulation and IL-8 production. **d** SRGN was mainly expressed in tumour cells while CD44 was detected in fibroblasts. **e** CD44 was mainly express in CAFs (red arrow) and some lymphocytes (yellow arrow) in gastric cancer. **f** c-Myc levels in CAFs were significantly higher than that in NFs. **g**, **h** Treatment with recombinant SRGN (rSRGN, 50 ng/ml) could increase c-Myc and KDM5B expression as well as IL-8 production in Hs738 cells, but addition of Angstrom6, a CD44 inhibitor, could suppress these effects (**i**, **j**). (***P < 0.001).

As reported previously, treatment with CMs from tumour cells activated Hs738 cells (Fig. 4a), and enhanced IL-8 levels (Fig. 4b), and KDM5B levels were increased accordingly (Fig. 4a). Importantly, the addition of rabbit anti-SRGN antibody inhibited KDM5B up-regulation and IL-8 production induced by treatment with the CMs of tumour cells (Fig. 4b, c). SRGN usually effects *via* the receptor CD44 [23]. We subsequently examined the expression of SRGN and CD44 in cell line, and found that SRGN was mainly expressed in tumour cells, whereas CD44 was detected in fibroblasts (Fig. 4d). IHC assays of stomach cancer tissues also showed that SRGN was mainly expressed in tumour cells (Fig. 3d), and CD44 tended to be expressed in CAFs and some lymphocytes (Fig. 4e). c-Myc has been shown to transcriptionally regulate KDM5B expression [24]. We found that c-Myc levels in CAFs were significantly higher than those in NFs (Fig. 4f), which was consistent with KDM5B expression (Fig. 1c). Treatment with recombinant SRGN increased c-Myc and KDM5B expression, as well as IL-8 production in Hs738 cells (Fig. 4g, h). Furthermore, addition of Angstrom6, a CD44 inhibitor, suppressed these effects (Fig. 4i, j). Collectively, these results suggested that tumour cell-derived SRGN promotes IL-8 expression in CAFs *via* the c-Myc/KDM5B pathway.

### TANs upregulate SRGN expression in gastric cancer cells *via* REG4

**Fig. 5:**
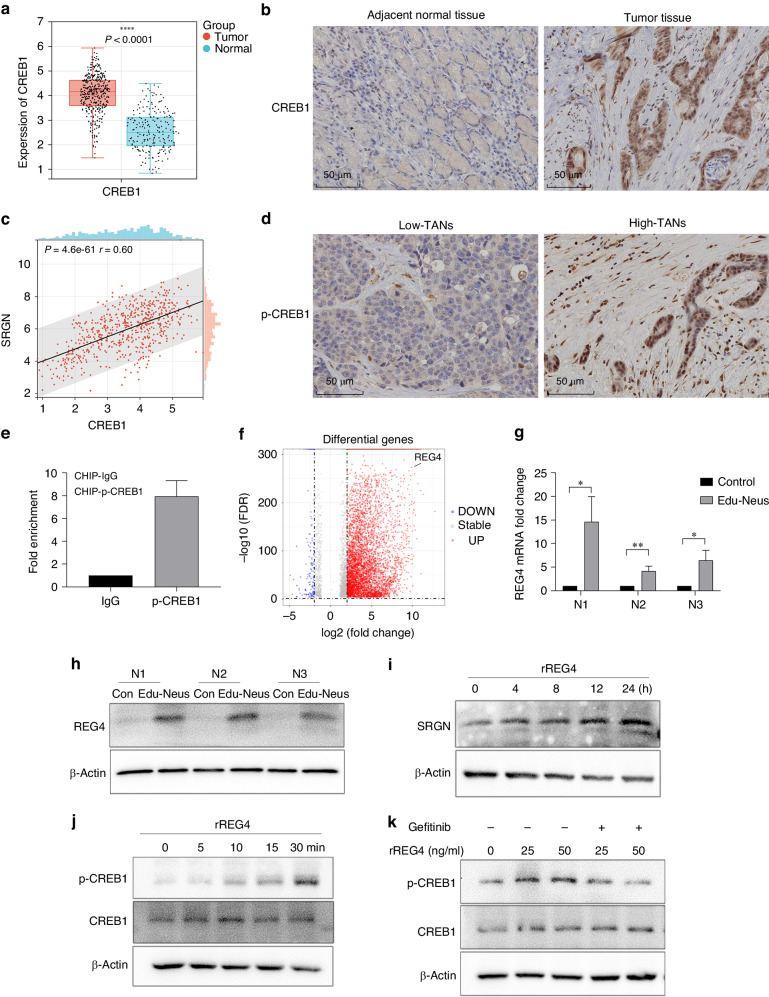
TANs upregulate SRGN expression in gastric cancer cells *via* REG4. **a** Analyses with TCGA database showed that CREB1 level was significantly higher in gastric cancer tissues than in normal mucosae (*P* < 0.001), and IHC assays with human stomach tumour tissues obtained similar results (**b**). **c** SRGN levels relate positively to CREB1 levels in gastric cancer. **d** CREB1 was highly activated (p-CREB1) in High-TANs group than in Low-TANs group. **e** CHIP-qPCR analysis showed that p-CREB1 could bind to the SRGN promoter region. **f** REG4 is one of the most significantly upregulated genes in Edu-Neus, which was verified by qPCR (**g**) and western blotting analysis (**h**). **i**, **j** Recombinant REG4 (rREG4) could activate CREB1 and upregulate the expression of SRGN in tumour cells, and Gefitinib, an EGFR inhibitor, could neutralise the effects of rREG4 (**k**). (*P < 0.05; **P < 0.01).

Next, we explored how neutrophils promote SRGN secretion by tumour cells. CREB1 has been reported to be the regulator of SRGN transcription [25]. Analyses using TCGA database showed that CREB1 levels were significantly higher in gastric cancer tissues than in normal mucosae (*P* < 0.001) (Fig. 5a), which was confirmed by IHC assays with human stomach tumour tissues (Fig. 5b). Bioinformatics analyses also showed a positive correlation between SRGN and CREB1 in gastric cancer (*P* < 0.001) (Fig. 5c). IHC assays with clinical specimens indicated that CREB1 was highly activated (p-CREB1) in the high-TANs group compared to that in the low-TANs group (Fig. 5d), which was consistent with SRGN (Fig. 3d). Furthermore, ChIP-qPCR analysis showed that p-CREB1 bound to the SRGN promoter region and regulated its transcription (Fig. 5e).

In our previous study, we showed that the interaction between neutrophils and tumour cells, namely educated-neutrophils (Edu-Neus) by tumour cells, is required for TANs-mediated tumour cell invasiveness [14]. Importantly, among the multiple differentially expressed genes (DEGs) in Edu-Neus, REG4 was one of the most significantly upregulated genes (Fig. 5f), as verified by qPCR and western blot analysis (Fig. 5g, h). Further experiments showed that recombinant REG4 (rREG4) activated CREB1 and upregulated SRGN expression in tumour cells (Fig. 5i, j). REG4 has been reported to functions *via* its receptor, EGFR [26]. Gefitinib, an EGFR inhibitor, neutralised the effects of rREG4 on SRGN expression and CREB1 activation (Fig. 5k). Collectively, these results suggest that TANs-derived REG4 upregulates SRGN in gastric cancer cells.

## Discussion

In this study, we unveiled the mechanisms underlying IL-8 production by CAFs in gastric cancer, and explored the important roles of the comprehensive crosstalk between tumour cells and stromal cells as CAFs and TANs *via* soluble molecules.

IL-8 has been demonstrated to be involved in tumour progression, and to be positively associated with larger tumour sizes, advanced stages, and poor prognoses in many cancer types [27–29]. In malignant gliomas, Liu et al. reported that IL-8 blockade can reshape the glioma TME from pro-tumour to antitumor status by eliminating both myeloid-derived suppressor cells (MDSCs) and mast cells and by repopulating tumour-associated macrophages (TAMs), resulting in an overall antitumor immune response in synergy with immune checkpoint blockade (ICB) therapy [30]. Xu et al. demonstrated that the prostate cancer cell-derived exosome IL-8 harms tumour-infiltrating CD8^+^ T cells by disturbing glucolipid metabolism, which fosters immune evasion [31]. In non-small cell lung cancer (NSCLC) cells, YY1 binds directly to the promoter region of IL-8 and transcriptionally activates IL-8 expression, resulting in tumorigenesis and EGFR-TKIs resistance [32]. Recently, Ma et al. reported that the IL-8-FAK-IL-8 positive feedback loop promotes the proliferation and migration of gastric cancer cells, leading to the development of gastric cancer peritoneal dissemination [33]. In our previous studies, we showed that IL-8 plays important roles in tumour invasion, metastasis, immunosuppression, and chemoresistance in gastric cancer [9–13]. However, cells that produce IL-8 in the TME remain elusive. Accumulating evidence has shown that IL-8 is generated by several cell types in the TME. Liu et al. showed IL-8 can be derived from tumour cells, myeloid cells, and a distinct subpopulation of CD4^+^ T cells in malignant gliomas [30] Even in gastric cancer, different cell origination of IL-8 has been reported. IL-8 is secreted by gastric cancer-associated mesenchymal stem cells (GC-MSCs) and is crucial for their oncogenic function [34]. Lin et al. reported that IL-8 is predominantly secreted by TAMs, which contributes to the immunosuppressive microenvironment by inducing PD-L1^+^ macrophages in gastric cancer [35]. However, our previous reports [11–13] and the current study using multiplex-immunofluorescence (MxIF) assays clearly demonstrated that IL-8 is mainly produced by CAFs in gastric cancer. In this study, we identified the transcription factor, KDM5B, in CAFs. KDM5B also acts as an H3K4 demethylase [15, 16]. Herein, we showed that H3K4 demethylase activity is also involved in IL-8 regulation in CAFs, but its detailed role requires further research. Furthermore, we demonstrated the reason for the inability to produce IL-8 in tumour cells, although KDM5B was present in tumour cells, which was attributed to competitive binding by a compromised tumour suppressor gene, RB1, in tumour cells.

In this study, we investigated the mechanisms underlying KDM5B up-regulation in CAFs. We found that tumour cell-derived SRGN increased KDM5B expression in CAFs *via* interacting with its receptor CD44. SRGN is the first proteoglycan (PG) whose core protein has been cloned and sequenced, and is also the most important intracellular proteoglycan to date. SRGN consists of an 18-kDa core protein and several glycosaminoglycan (GAG) chains [36]. Proteoglycans (PGs) play a crucial role in tumour progression [37]. SRGN has been revealed to be highly expressed in multiple cancer types, including breast cancer, colon cancer, lung cancer and nasopharyngeal cancer [38]. SRGN plays crucial roles in the interaction between tumour cells and stromal cells and in the modulation of tumour immune microenvironment. Tanaka et al. reported that SRGN can increase the migratory and invasive properties of tumour cells and fibroblasts in lung adenocarcinoma by regulating the expression of PD-L1 and pro-inflammatory cytokines such as IL-6, IL-8, and C-X-C motif chemokine 1 [39]. Bouris et al. showed that SRGN promotes proliferation, migration, and invasion of breast cancer cells by increasing the secretion of IL-8 and triggering IL-8/CXCR2 downstream signalling cascades [40]. SRGN mainly functions by binding to CD44 [23]. Zhu et al. reported that SRGN promotes tumour invasion and metastasis in oesophageal squamous cell carcinoma *via* the SRGN/MDK/CD44 complex to activate the ERK pathway, stabilise c-Myc, and upregulate the secretion of matrix metalloproteinases [36].

**Fig. 6:**
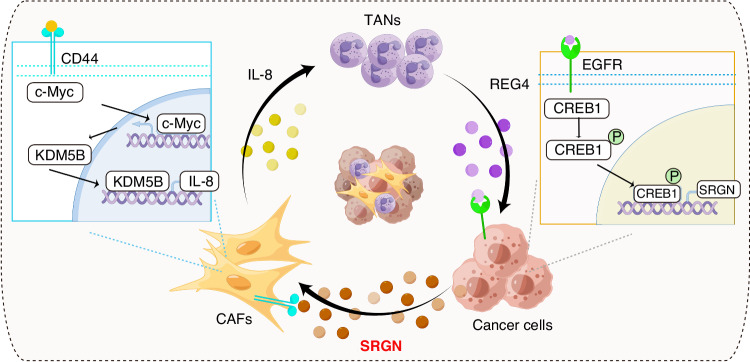
Schematic representation of the mechanism for the preferential production of IL-8 by CAFs in gastric cancer. Tumour cell-derived SRGN upregulates KDM5B expression in CAFs *via* interacting with its receptor CD44 and increases IL-8 production in CAFs. IL-8 further recruits neutrophils into the TME. The infiltrated neutrophils, namely, tumour-associated neutrophils (TANs), upregulate SRGN expression in gastric cancer cells *via* REG4. Thus, the SRGN-IL-8-TANs-SRGN loop facilitates tumour progression.

Furthermore, we ascertained the modulation of SRGN expression in the gastric cancer microenvironment. As a major neutrophil chemokine, IL-8 levels in the TME are associated with increased TANs in gastric cancer. TANs educated by tumour cells have been shown to be involved in gastric cancer progression [14, 41]. In this study, we showed that REG4 was upregulated in TANs. REG4 activates CREB1 by interacting with its receptor, EGFR, in tumour cells, resulting in increased SRGN expression. Collectively, we explored a SRGN-IL-8-TANs-SRGN positive feedback loop in gastric cancer (Fig. 6). This loop may promote the continuous release of SRGN and IL-8 into the TME of gastric cancer, thereby facilitating tumour progression.

Our study also suggests that SRGN and CD44 may be potential targets for cancer therapy, as He et al. mentioned [42]. We showed that SRGN knockdown or CD44 inhibition decreases IL-8 production in gastric cancer cells. IL-8 clearance has been shown to be benefitial for cancer patients [30, 43]. Further, in vivo studies are required to evaluate the therapeutic roles of SRGN and CD44.

In summary, the current study revealed the mechanisms underlying the preferential production of IL-8 by CAFs in gastric cancer. This facilitates the understanding of the critical roles of the interaction between tumour cells and stromal cells in gastric cancer progression. This study provides SRGN and CD44 as potential therapeutic targets to reduce IL-8 production in gastric cancer.

### Supplementary information

Supplementary Materials

## Data Availability

The datasets used and/or analysed during the current study are available from the corresponding author upon reasonable request.
