# Supplementary material for: Tumour cell-derived serglycin promotes IL-8 secretion of CAFs in gastric cancer
Source: Br J Cancer. 2024 Jun 11;131(2):271–82. doi: 10.1038/s41416-024-02735-2 (PMC11263384; doi:10.1038/s41416-024-02735-2)
Supplement: Supplementary file 1 — Supplementary Materials [file 41416_2024_2735_MOESM1_ESM.pdf]

Supplementary Materials

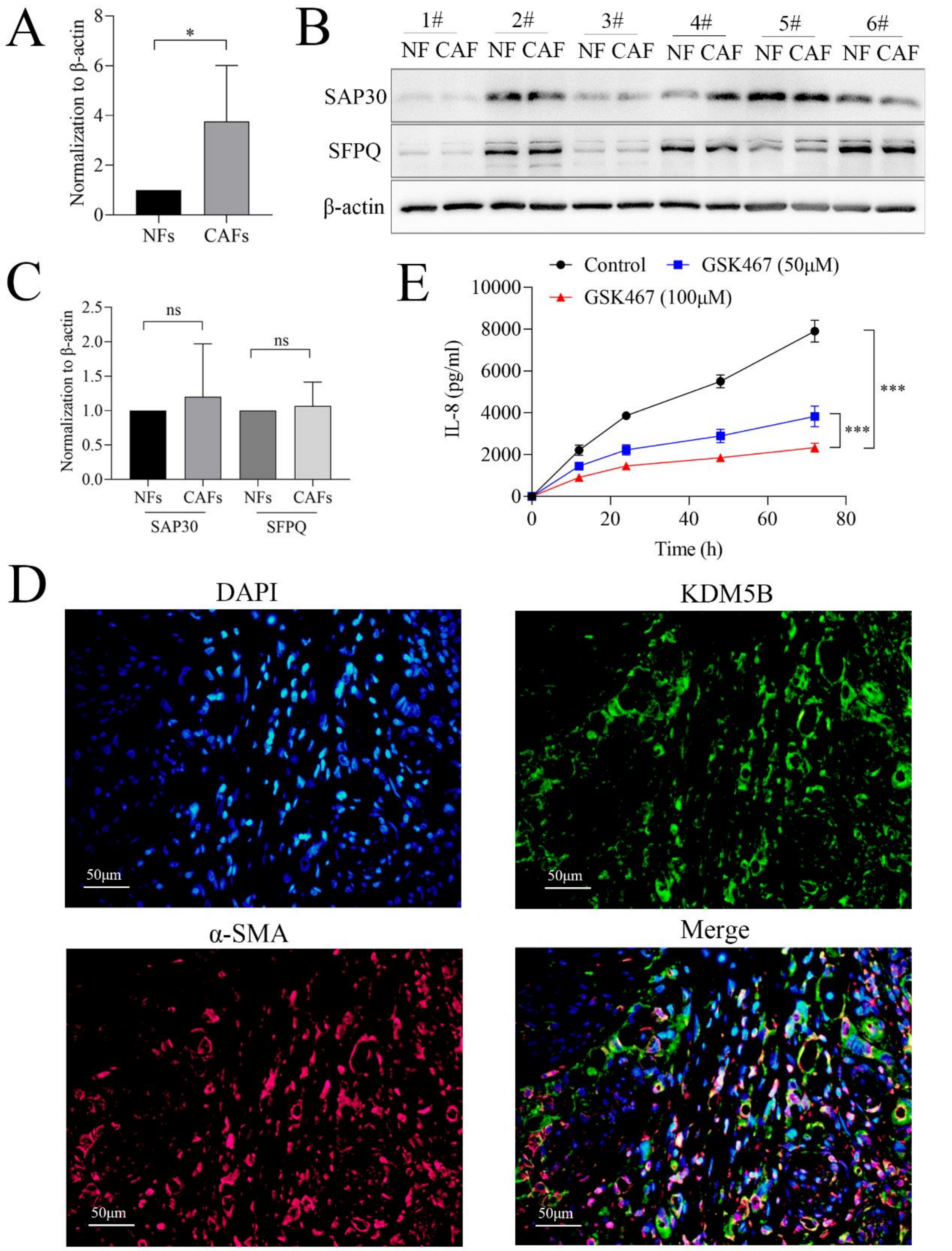

Figure s1. KDM5B is responsible for IL-8 expression in CAFs. (A) KDM5B was up-regulated in CAFs with regard to NFs. (B, C) The expressions of SAP30 and SFPQ showed no significant difference between in CAFs and NFs. (D)

Immunofluorescence analysis of gastric tumor tissues showed that KDM5B (green) was mainly expressed in most of CAFs (α-SMA, red). (E) Treatment with KDM5B inhibitor, GSK467, resulted in the reduction of IL-8 production in CAFs. (ns, no significance; *, $P < 0.05$; ***, $P < 0.001$)

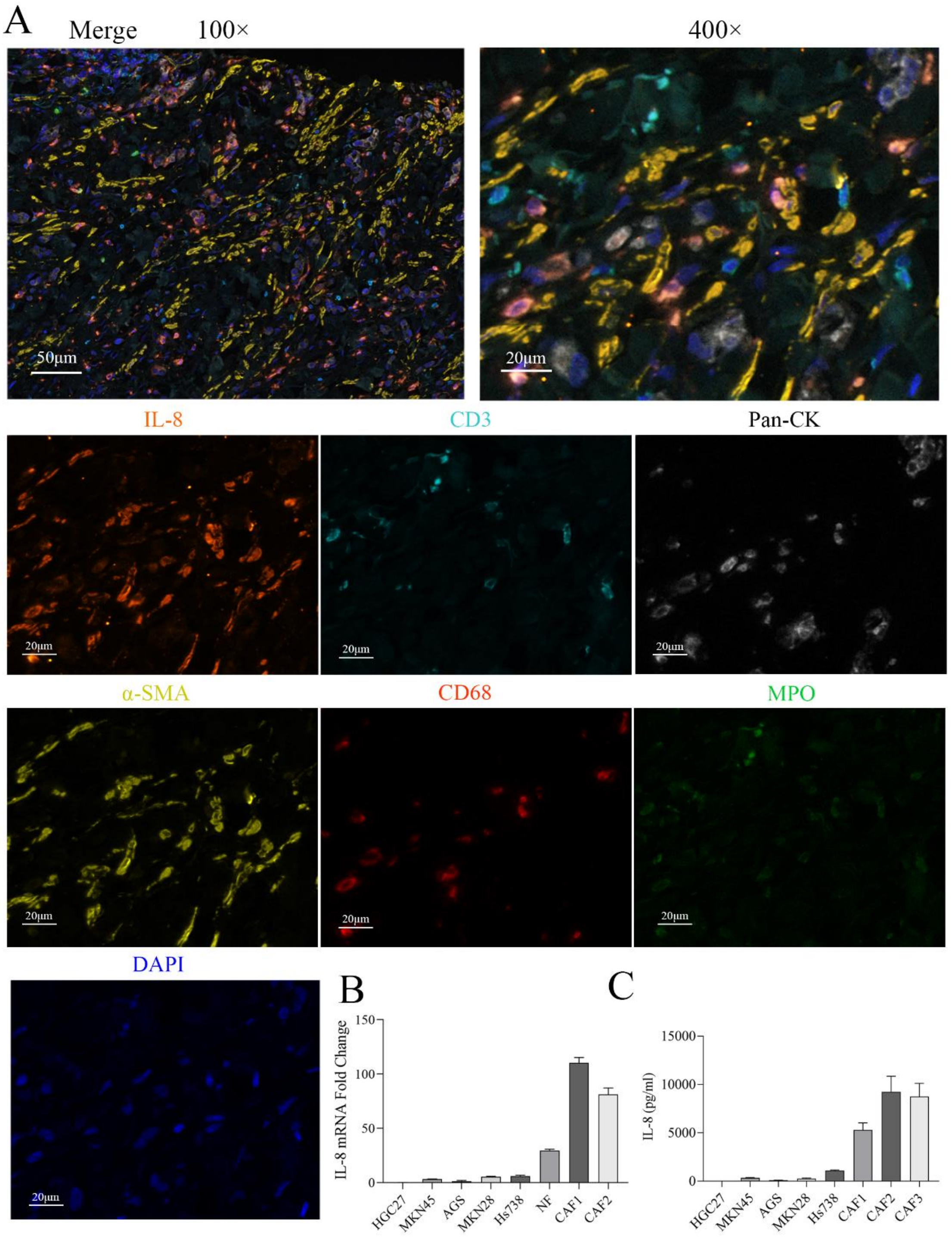

Figure s2. IL-8 was mainly expressed in CAFs in gastric cancer. (A) MxIF staining of gastric cancer tissues showed that IL-8 was mainly expressed in α-SMA positive cells. (B) Assays with qPCR showed that IL-8 was mainly expressed in CAFs. (C) IL-8 level was much higher in CMs of CAFs.

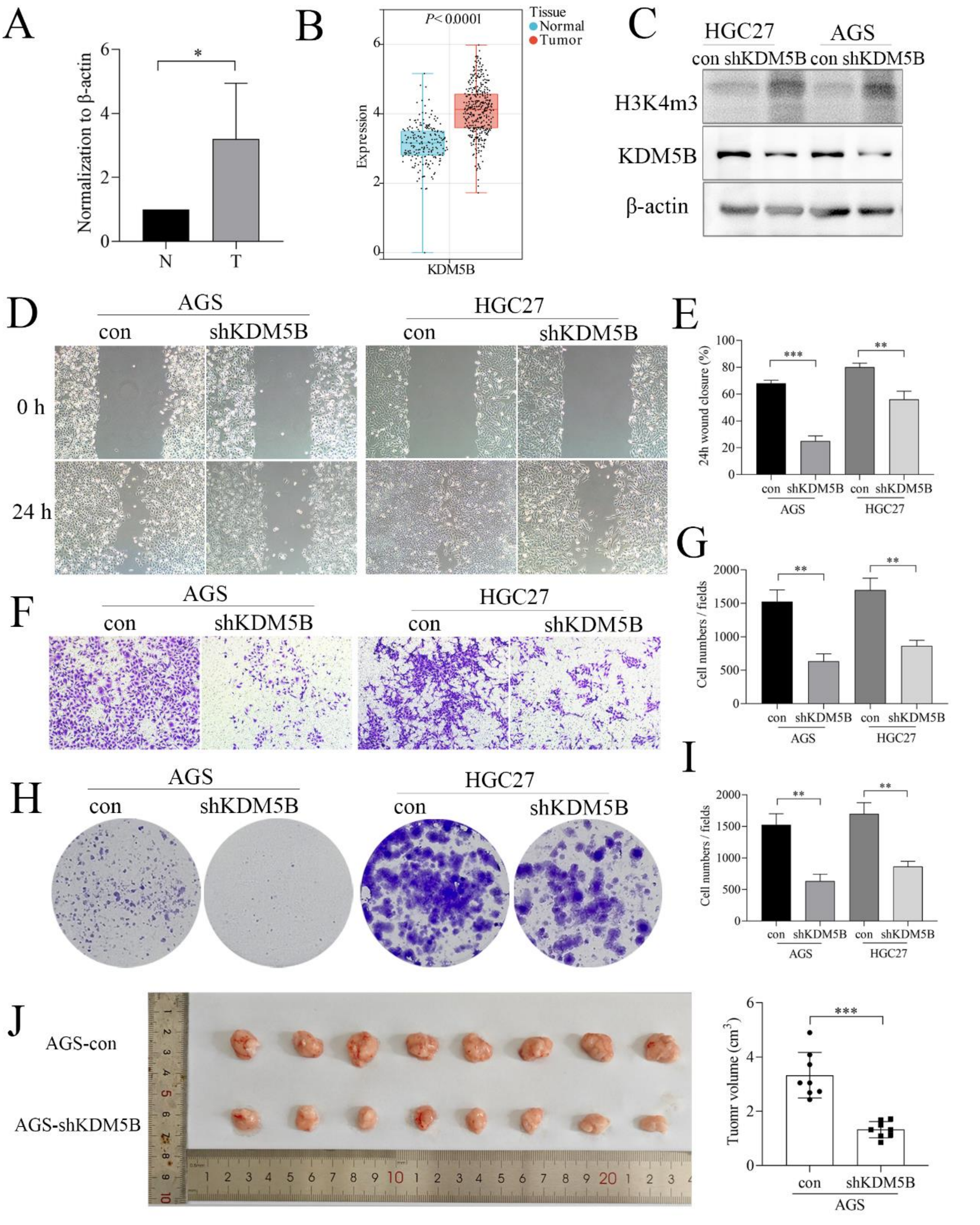

Figure s3. KDM5B is closely related to gastric cancer progression. (A) KDM5B level in stomach cancer tissues was much higher than in adjacent normal mucosa tissues. (B) Analyses with TCGA database showed that KDM5B levels in gastric cancer tissues are significantly higher than in adjacent normal tissues. (C) KDM5B knockdown with shRNA significantly increased the H3K4 tri-methylation level in HGC27 and AGS cells.

Wound-healing (D, E), transwell (F, G), and clone formation assays (H, I) showed that KDM5B knockdown significantly suppressed the proliferation, invasion and migration abilities of gastric cancer cells. (J) The in vivo study in Balb/c nude mice showed that KDM5B silence with shRNA suppressed tumor cell proliferation (*, $P < 0.05$; **, $P < 0.01$; ***, $P < 0.001$)

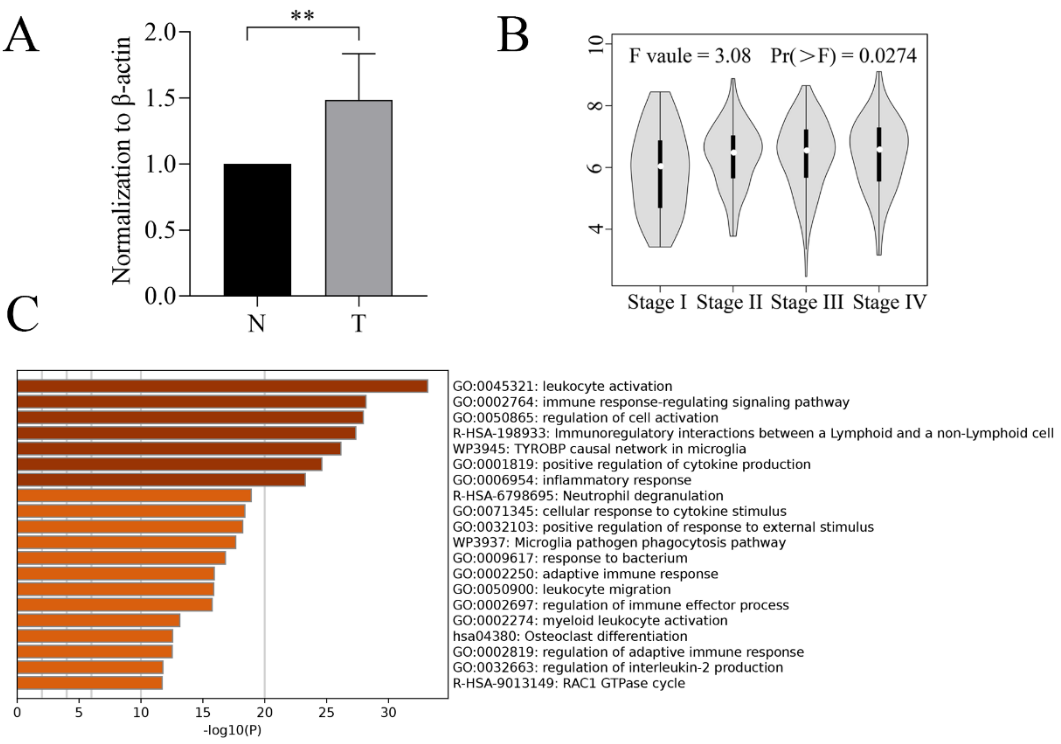

Figure s4. The bioinformatics analysis of SRGN. (A) SRGN was up-regulated in gastric cancer tissues than in adjacent normal mucosa tissues. (B) Analyses with TCGA database showed SRGN levels in tumor tissues are closely associated with clinical stage. (C) Bioinformatics analyses showed the enrichment pathways of SRGN-related genes, which main enriched in neutrophil activation, immune regulation and inflammatory response. (**, $P < 0.01$)

Table s1. Four primer pairs of IL-8 and SRGN promoter region used in qPCR analyses

	CHIP / qPCR promoter region	
IL-8	1F	CAGTGTGGGCAAATTCACTG
	1R	GGTTCCAACTCTGGTTCAGC
	2F	GCAAATTCACTGCTCTGTCG
	2R	GGTTCCAACTCTGGTTCAGC
	3F	GCAAATTCACTGCTCTGTCG
	3R	CACTGGAGCTGCTTGGTTG
	4F	TGCACTGTGTTCCGTATGCT
	4R	AATGCCTGGGTTACACTGAA

SRGN	1F	TCAAATGCGTTTGCTTGAAC
	1R	CCGGAAAGTTTTCCACTGAG
	2F	GGGTTGGTTACTGTGCTTGG
	2R	TTGCCCTTGTGGCATATACA
	3F	CCACAAGGGCAAGGATGTAT
	3R	CAATAAAGGCCAGGTGCTGT
	4F	TGGAGCCTTAGTCCTCCAGA
	4R	GCCCAGCAAACAGTTTCTTC
